# Implementation of a complex intervention to reduce hospitalizations from nursing homes: a mixed-method evaluation of implementation processes and outcomes

**DOI:** 10.1186/s12877-022-02878-y

**Published:** 2022-03-12

**Authors:** Kornelia Basinska, Franziska Zúñiga, Michael Simon, Sabina De Geest, Raphaëlle Ashley Guerbaai, Nathalie I. H. Wellens, Dunja Nicca, Thekla Brunkert

**Affiliations:** 1 Nursing Science (INS), Department Public Health (DPH), Faculty of Medicine, University of Basel, Bernoullistrasse 28, 4056 Basel, Switzerland; 2 Inselspital Bern University Hospital, Nursing Research Unit, Freiburgstrasse 4, 3010 Bern, Switzerland; 3 Department of Public Health and Primary Care, Academic Centre for Nursing and Midwifery, KU Leuven, Leuven, Belgium; 4 Department of Public Health and Social Affairs of the Canton of Vaud, Lausanne, Switzerland; 5 La Source School of Nursing, HES-SO University of Applied Sciences and Arts Western Switzerland, Lausanne, Switzerland; 6 Department of Public & Global Health, Epidemiology, Biostatistics and Prevention Institute, University of Zurich, Zurich, Switzerland; 7 University Department of Geriatric Medicine FELIX PLATTER, Basel, Switzerland

**Keywords:** Implementation science, Intervention, Nursing homes, Uptake

## Abstract

**Background | objective:**

To evaluate the implementation of three intervention elements to reduce hospitalizations in nursing home residents.

**Design:**

Convergent mixed-method design within a hybrid type-2 effectiveness-implementation study.

**Setting:**

Eleven nursing homes in the German-speaking region of Switzerland.

**Participants:**

Quantitative data were collected from 573 care workers; qualitative data were collected from 108 care workers and the leadership from 11 nursing homes.

**Intervention:**

Three intervention elements targeting care workers were implemented to reduce unplanned hospitalizations: (1) the STOP&WATCH instrument for early recognition of changes in resident condition; (2) the ISBAR instrument for structured communication; and (3) specially-trained INTERCARE nurses providing on-site geriatric support. Multifaceted implementation strategies focusing both on the overall nursing home organization and on the care workers were used.

**Methods:**

The quantitative part comprised surveys of care workers six- and twelve-months post-intervention. The intervention’s acceptability, feasibility and uptake were assessed using validated and self-developed scales.

Qualitative data were collected in 22 focus groups with care workers, then analyzed using thematic analysis methodology. Data on implementation processes were collected during implementation meetings with nursing home leadership and were analyzed via content analysis. Findings were integrated using a complementary approach.

**Results:**

The ISBAR instrument and the INTERCARE nurse role were considered acceptable, feasible, and taken up by > 70% of care workers. The STOP&WATCH instrument showed the lowest acceptance (mean: 68%), ranging from 24 to 100% across eleven nursing homes. A combination of factors, including the amount of information received, the amount of support provided in daily practice, the users’ perceived ease of using the intervention and its adaptations, and the intervention’s usefulness, appeared to influence the implementation’s success. Two exemplary nursing homes illustrated context-specific implementation processes that serve as either barriers or facilitators to implementation.

**Conclusions:**

Our findings suggest that, alongside the provision of information shortly before intervention start, constant daily support is crucial for implementation success. Ideally, this support is provided by designated and trained individuals who oversee implementation at the organizational and unit levels. Leaders who seek to implement interventions in nursing homes should consider their complexity and their consequences for workflow to optimize implementation processes accordingly.

**Trial registration:**

This study was registered at clinicaltrials.gov (NCT03590470) on the 18/06/2018.

**Supplementary Information:**

The online version contains supplementary material available at 10.1186/s12877-022-02878-y.

## Introduction

Unplanned hospitalizations of nursing home (NH) residents are frequent; however, up to 60% of these are avoidable [1]. In addition to the obvious financial burdens these events entail, they put residents at significant risk for complications including infections, falls, and delirium, which commonly reduce their quality of life by precipitating physical and cognitive deterioration [2, 3].

Studies describe several factors, which contribute to a great number of unplanned nursing home transfers [4]. These factors include delayed recognition of resident deteriorating conditions due to lack of on-site geriatric expertise, a lack of timely assessment and timely management, and non-effective communication between care providers [4]. Therefore, to address the complexity of and reduce the risk for unplanned hospitalizations complex interventions combining multifactorial approaches have proven successful [4]. These include early symptom recognition, structured nurse-physician communication, and on-site clinical nursing support, which showed reductions in unplanned hospitalizations [5, 6]. So far, these complex interventions were mainly tested in the United States (US). However, NHs in other countries with similar problems related to unplanned hospitalizations might also benefit from them [7–10]. Successful translation of evidence into another health care settings requires intervention tailoring based on the local NH needs and resources to make them feasible and sustainable [11, 12]. For example, the availability of electronic documentation will drive the process of how communication instruments are used and documented to enhance feasibility. Availability of advanced practice nurses (APNs) will determine if NHs can hire APNs to bring in geriatric expertise, as some US studies have demonstrated or if alternative roles such as the of registered nurses in expanded roles are feasible [13]. Another aspect supporting implementation of complex interventions is the tailoring of implementation strategies with regard to local hindering and facilitating factors [14].

Barriers and facilitators to interventions’ implementation in health care settings have been well-studied [15]. Those most frequently reported include leadership, communication and the target users’ knowledge, beliefs and general attitudes concerning an intervention [15]. A comprehensive contextual analysis preceding implementation efforts and continuous stakeholder involvement can provide valuable insights to facilitate tailoring of interventions and implementation strategies [14].

Likewise, a retrospective evaluation of the implementation from a user’s perspective can help discern the various conditions’ influences on success [11, 12]. Central indicators of a successful implementation are implementation outcomes. In the early phase of an implementation effort, acceptability, defined as relevant stakeholders’ perceptions that an intervention is agreeable or satisfactory., and feasibility, the degree to which target users regard an intervention as suitable and practical for uptake in a given context, are essential conditions to facilitate the intervention’s uptake (defined as intention to or initial adoption of an intervention) [12]. Knowledge gained from a comprehensive evaluation of implementation processes and outcomes can inform future efforts to spread interventions to other NHs and thus increase the overall impact for NH residents.

This analysis is part of an implementation science hybrid type 2 effectiveness-implementation study [16] named “Nurse-led model of care in Swiss nursing homes: improving interprofessional care for better resident outcomes” – INTERCARE” [17]. INTERCARE is currently testing a complex intervention to reduce unplanned hospitalizations from Swiss NHs [17]. Reporting from the care workers´ perspective of implementation effectiveness, this article will concentrate on the three intervention elements used by the participating NHs’ care workers. All three interventions were developed and tailored to the Swiss context, based on a contextual analysis of nurse-led care models in Swiss NHs and stakeholder feedback [17, 18]. These include (1) the STOP&WATCH instrument for nurse aids for early recognition of changes in residents’ condition and their communication [7], (2) the ISBAR instrument to structure communication between registered nurses (RNs) / licensed practical nurses (LPNs) with physicians [7], and (3) the INTERCARE nurses, specially designated roles for RNs providing ongoing on-site clinical support [18]. The INTERCARE intervention’s effectiveness regarding the reduction of unplanned hospitalizations has been evaluated elsewhere [19]. Both instruments STOP& WATCH and ISBAR were adapted from the Interventions to Reduce Acute Care Transfers (INTERACT) quality improvement intervention [7].

### Objectives

The overall aim of this mixed-method study is to evaluate the implementation of three intervention elements from the intervention users’ perspective across 11 NHs. The study focuses on three main objectives: (1) to assess degree of acceptability, feasibility, and uptake of the intervention elements from the intervention users` perspective (quantitative aim); (2) to describe the implementation process in two NHs while exploring implementation barriers and facilitators from the intervention users´ perspective (qualitative aim); and (3) to generate an understanding of inter-NH differences in the various intervention elements’ acceptability, feasibility and uptake based on the qualitative data from interventions users`(mixed-method aim).

## Methods

### Design

This study used a convergent mixed-method design to evaluate the implementation of three intervention elements within the INTERCARE study [20].

### INTERCARE intervention

The INTERCARE intervention was implemented using a non-randomized stepped-wedge design in a purposeful sample of eleven NHs in Switzerland’s German-speaking region [17]. Depending on when participating NHs enrolled, the intervention lasted between 12 and 18 months: the first NH started in September 2018 and the last in February 2019. Data collection finished for all NHs in February 2020. The three intervention elements evaluated in this study focused on the NH care workers: (1) The STOP&WATCH instrument aimed to help early recognition of common but nonspecific changes in resident conditions before they become severe. This checklist identifies potential “gray zones” and therefore serves as support for regular follow-up and interprofessional communication (Supplementary file 3); (2) In the next step, the ISBAR instrument guides LPNs and RNs in structuring communication of the observed changes to physicians. The “Introduction, Situation, Background, Assessment, Recommendation” (ISBAR) structure enhances efficient and unambiguous information transfer, an important step in interprofessional collaboration (Supplementary file 4.); and (3) The INTERCARE nurse. INTERCARE nurses are trained RNs with at least 3 years’ NH experience are recruited and employed by each NH to deliver at least 24 h/week on-site clinical care, coaching and support per 80 beds [17]. The competencies of the INTERCARE nurse were systematically developed in a three-step process including (1) critical literature review, (2) case studies, and (3) stakeholders rating, and as described elsewhere [18]. Additionally, the INTERCARE nurse participated in a standardized educational programme [18].

A detailed description of the intervention elements is presented according to the Action, Actor, Context, Target, Time (AACTT) framework in Table 1 [21]. A detailed description of the INTERCARE study, including its intervention elements and eligibility criteria for NHs and residents, is included in the study protocol [17].

**Table 1:** Description of the intervention elements based on the Action, Actor, Context, Target, Time (AACTT) Framework [17]

Action	Actor	Context	Target	Time
**STOP&WATCH**				
Identifying, documenting any change in resident conditions and passing the information to the responsible nurse	Nurse aide	Units in the nursing home	Residents living in a nursing home	During resident's care provision
Evaluating reported resident changes in condition and perform adequate assessment if necessary	Registered nurse Licensed practical nurse	Units in the nursing home	A resident with changes in condition	After receiving a STOP&WATCH
**ISBAR (Introduction, Situation, Background, Assessment, Recommendation)**				
Communication about residents’ care or change in residents’ health status	Registered nurse Licensed practical nurse	Remote and in-person communication about resident	Physicians	Every time physician is contacted
**INTERCARE nurse**				
Coaching nursing staff and supporting in resident care	INTERCARE nurse	When asked / when noticing complex situations	Nurse aid Registered nurse Licensed practical nurse	During bedside care
Supporting or managing conversations with residents/relatives	INTERCARE nurse	When asked / when noticing complex situations	Registered nurse Licensed practical nurse	During bedside care / relatives visit or phone calls
Supporting in decision making about residents' care"	INTERCARE nurse	When asked / when noticing complex situations	Registered nurse Licensed practical nurse	During the decision-making process
Supporting in preparation for physician visit/communication	INTERCARE nurse	When asked / when noticing complex situations	Registered nurse Licensed practical nurse	Before scheduled physician visit/ communication

This study has been approved by ethic committee of “Ethikkommission Nordwest und Zentralschweiz” (EKNZ, 2018–00501) and was registered on 18/06/2018 at clinicaltrials.gov (NCT03590470). The recruitment is completed.

### Implementation strategies

All strategies were defined based on a contextual analysis guided by the Consolidated Framework for Implementation Research (CFIR) [22] and stakeholders’ input in the development phase of INTERCARE [17]. The CFIR is a meta-theoretical implementation science framework that provides a list of factors that are potentially influencing for implementation [22]. An overview and operationalization of implementation strategies following the “Expert Recommendations for Implementing Change” (ERIC) are presented in Table 2 [14]. Throughout the study period, to support and reflect on the intervention elements’ implementation, we conducted implementation meetings with the NHs and telephone calls with INTERCARE nurses. Further, throughout the study, we employed three implementation strategies for the INTERCARE nurse and two more for the STOP&WATCH and ISBAR components. Participating NHs could also appoint champions to support the INTERCARE nurses in planning, monitoring, and evaluating both instruments’ unit-level implementation.

**Table 2:** Overview of implementation strategies [10]

Focus	Implementation strategy	Operationalization
Overall implementation support	Provide ongoing consultation	Bi-monthly implementation meetings (2 h) between the nursing home leadership (incl. Nursing directors, INTERCARE nurses) and the research group to support and reflect on the intervention elements’ implementation, and to provide information. Structured meeting notes were collected to capture implementation processes and relevant experiences.
		Bi-weekly implementation telephone calls (1 h) between INTERCARE nurses and the study coordinator to support, reflect and address the implementation of the role, STOP&WATCH, and ISBAR and to identify problems. During the phone calls structured notes were collected to capture implementation processes and relevant experiences.
INTERCARE nurse	Conduct ongoing training	Provision of education and training for INTERCARE nurses (approximately 390 h) throughout the study. Topics comprised: clinical skills (e.g., comprehensive geriatric assessment), leadership, communication, quality improvement and information about the intervention elements (e.g., STOP&WATCH, ISBAR).
	Develop and distribute educational materials	Research group posted on an online educational and training platform different educational material, e.g., readings, videos were posted for the INTERCARE nurses to support implementation.
	Make training dynamic	The training for INTERCARE nurses comprised blended learning with e-learning and in-person education approaches to support learning.
STOP&WATCH and ISBAR	Create new clinical teams	The INTERCARE nurses constituted a new member of the interprofessional care team and in this role their role is to facilitate the implementation of ISBAR and STOP&WATCH in the nursing home. They are responsible for planning, monitoring, evaluating the implementation in the nursing homes.
	Develop and distribute educational materials	At the start of the implementation staff handouts, flyers, posters, PowerPoint presentations, and pocket versions of STOP&WATCH and ISBAR were distributed to the care workers.
Optional strategy	Identify and prepare champions	Nursing homes received implementation guidelines with the suggestion to appoint and train champions, i.e., local facilitators, on each unit to support the INTERCARE nurses in planning, monitoring, and evaluating both instruments’ unit-level implementation. The INTERCARE nurse prepared the champions.

*Note:* ISBAR*:* Introduction, Situation, Background, Assessment, Recommendation

### Quantitative part

#### Sample and data collection

Quantitative data were collected at two time points in each of the eleven NHs participating in the INTERCARE study—counting from the start of the study in each location, the first (T1) was after 6 months, (between April and September 2019), the second (T2) after 1 year (between October 2019 and February 2020). Surveys of care workers, including registered nurses (RNs), licensed practical nurses (LPNs), and nurse aides. Participants were included if they were currently working in direct resident care, had been employed for at least 3 months at the time of data collection, and were sufficiently fluent in German to understand the survey questions. In each NH, local coordinators distributed the questionnaires. Return of the questionnaire implied informed consent. To ensure confidentiality, a pre-stamped envelope was provided with each questionnaire.

#### Measures

The three intervention elements’ acceptability and feasibility were measured 6 months (T1) after intervention start [12]. We used two validated scales, each using four five-point items (range: 1–5): The *Acceptability of Intervention Measure* (AIM) and the *Feasibility of Intervention Measure* (FIM) [23]. Cronbach’s alphas for these scales were 0.96 both for acceptability and for feasibility. Care workers rated their items from *do not agree* (1 point) to *agree* (5 points), with higher scores indicating greater acceptability (e.g., *I like the INTERCARE nurse*) or feasibility (e.g., *STOP&WATCH seems to be implementable*) [23]. To score the acceptability and feasibility scales, we summed the ratings and averaged them across the four items (possible range: 1–5). Lastly, to improve readability and comparability we divided the final averages into one dichotomous categorical variable [24].

The three intervention elements’ uptake data were collected 12 months (T2) after intervention start via self-developed items. As above, all items collected data via 5-point Likert-type scales (range: *never* (1)–*always* (5)). For each item, we assessed nurse aides’ uptake of STOP&WATCH (*I use STOP&WATCH when I notice a difference in a resident’s condition*) and of the INTERCARE nurse role (*When required I receive coaching and support for residents’ care*). To assess RNs’/LPNs’ uptake of ISBAR, we used a single item (*I use ISBAR when I contact a physician*). To assess the RNs’/LPNs’ uptake of the INTERCARE nurse, though, we used four items (e.g., “When required, I receive coaching and support in preparation for physician visits/communication”). All items are reproduced in in the Supplementary file 1.

To calculate the uptake of individual elements (i.e., STOP&WATCH, ISBAR, INTERCARE nurse from the nurse aids’ perspective), we created a dichotomous variable by combining the *never/seldom* and *sometimes/often/always* answer options for three items and calculated uptake based on the proportion of care workers who used each element *sometimes/often/always*.

To calculate the RNs’/LPNs’ uptake of the INTERCARE nurse, we first summed their 5-point ratings and averaged them across the four items before creating a dichotomous variable. Further items assessed these workers’ sociodemographic data (job title, sex, age, employment, working experience in nursing and in this NH, usual shift).

#### Data analysis

Descriptive statistics (percentages, means, and standard deviations) were calculated for sociodemographic variables. Based on the dichotomization described above, acceptability, feasibility, and uptake of these elements were assessed as proportions. We considered an intervention element as successfully implemented if ≥ 70% participants report that they accept it, find it feasible, and have taken it up [25]. To assess inter-NH outcome differences, we used the Chi-squared test, with significance set at *p* < .05. Records with more than 50% of data missing per scale were excluded from the analyses. Statistical analyses were conducted in R, version 1.1.463 [26].

### Qualitative part

#### Sample and data collection

This study’s qualitative part used data from two sources: structured notes from implementation meetings and telephone calls, and focus groups with care workers including registered nurses, licensed practical nurses and nurse aides in eleven NHs.

To acquire information on internal implementation process, we collected structured notes from implementation meetings with NH leadership and telephone calls with INTERCARE nurses in all 11 NHs (Table 2). During the bi-monthly implementation meetings with NH leadership in each NHs, the second author conducted semi-structured discussions with NH leadership to capture implementation process-relevant experiences. The study coordinator took structured notes, which the second author checked for accuracy after each implementation meeting. Additionally, the study coordinator took structured notes in bi-weekly implementation telephone calls with INTERCARE nurses.

We conducted focus groups interviews with RNs, LPNs and nurse aids in all 11 NHs 6 months after implementation start to describe the implementation barriers and facilitators (T1, between April and September 2019). Following the same inclusion criteria as for the quantitative part, the local INTERCARE nurses recruited convenience samples of care workers. Before each focus group interview, the INTERCARE nurse informed eligible staff about the study, distributed written study information, and obtained informed consent prior to the interviews. A German-language interview guide with open-ended questions was developed to explore barriers and facilitators to the intervention’s uptake and adapted to either focus group interview with nurse aids or LPNs/ RNs (e.g., What does it take for the STOP&WATCH/ISBAR instrument to be used on your unit?, What did you think about the role of the INTERCARE nurses at the beginning?).

All focus groups were moderated by the first author, a PhD student trained in qualitative methodology. A research assistant summarized the main topics to validate them during the focus groups. In each NH, two focus groups were conducted: one with nursing aides, one with RNs and LPNs together. The 22 interviews lasted 45–92 min (mean: 75 min) and were audio-recorded. For our analysis, eight were transcribed verbatim; the remaining 14 remained as audio files due to time and financial limitations.

#### Data analysis

To analyze structured notes on the implementation process from all NHs we used Mayring’s content analysis approach [27]. In the first step of the analysis inductive categories (e.g., implementation plan and process, implementation, evaluation, Table 5.) were developed based on the data. In the second step the notes, both from the NH implementation meetings and from the telephone calls with the INTERCARE nurses, were coded using developed categories by the first author separately for each NH [27]. In an iterative process, categories were discussed and refined in collaboration with the last author. To describe successful and unsuccessful approaches to the implementation process we used a deviance approach, focusing on two NHs where the quantitative phase yielded particularly high and low uptake results [28].

We used data from the focus groups with RNs, LPNs and nurse aids to explore care workers’ views and perceptions regarding barriers and facilitators encountered during the first 6 months of implementation of the intervention elements. The reflexive thematic analysis [29, 30] with a constructivist orientation [31] was used to analyze eight transcripts and 14 audio files [32] in their original language- The analysis was done by a qualitative research team comprised of three members: a PhD student in Nursing Science, a postdoctoral researcher and a senior researcher. The analyses were conducted using Maxqda Analytics Pro [33]. The analytical steps are presented in the Supplementary file 2.

### Data integration

To explain differences in implementation of the three intervention elements across NHs we used a complementarity approach [20, 34]. We first analyzed the quantitative and qualitative data separately, then integrated the data in two stages. First, based on the quantitative results, we selected two NHs: the one with the highest (NH2) intervention element uptake and the one with the lowest (NH11). Our aim was to describe and compare the most and least successful implementation processes. Secondly, during the interpretation stage, to examine the eleven NHs’ care workers’ differences concerning acceptability, feasibility and uptake, we contrasted the quantitative results for each intervention element (STOP&WATCH, ISBAR, INTERCARE nurse) with the qualitative themes and the data from the implementation process.

## Results

### Quantitative data

Overall, the sample included 573 care workers from eleven NHs (response rate 78%). Table 3 overviews the participating NHs’ and care workers’ sociodemographic characteristics.

**Table 3:** Characteristics of participating nursing homes and care workers

Nursing home characteristics	(***n*** = 11)
**Legal status**	**n (%)**
Privately funded	9 (81.8)
Publicly funded	2 (18.2)
**Location**	
Urban	8 (72.7)
Rural	2 (18.2)
Suburban	1 (9.1)
**Bed count**	**Median (IQR)**
All long-term beds	120 (114–161)
Number of beds participating in INTERCARE	88 (80–103)
**Physician model**	**n (%)**
NHs working with primary care physicians not employed by the nursing homes	4 (36.4)
NHs working with employment/contractual arrangements with physicians	3 (27.2)
NHs working with mixed physician models	4 (36.4)
**Care worker characteristics**	**(*n*** **= 573)**
**Professional group**	**n (%)**
Registered nurse^a^	135 (24)
Licensed practical nurse^2^	212 (37)
Nurse aide^c^	226 (39)
**Sex** (Female)**; n (%)**	475 (85)
**Age (mean;** SD (range))	41; 13.5 (17–67)
**Working experience**	**Mean (IQR)**
In nursing (years)	13.7 (5–20)
In this NH (years)	7.7 (2–11)
**Usual shift**	**(n /%)**
Day shift	183 (33.9)
Late shift	31 (5.7)
Night shift	48 (8.9)
Regular shift changes	277 (51.4)

^a^Registered nurse with 3–4-year education; ^b^ Licensed practical nurse with three years of education; ^c^ Nurse aide with 1–2 months of education or on the job training /short course

#### Acceptability and feasibility of intervention elements

Regarding acceptability and feasibility, we found inter-NH differences for all intervention elements. In NH 6 and NH 11, the overall acceptability values were below the threshold of 70% for all three elements (STOP&WATCH 57%/26%; ISBAR 68%/44%; INTERCARE nurse 67%/62%). In contrast, NH4 reached the highest acceptability, with rating of 100% for STOP&WATCH, 94% for ISBAR and 100% for the INTERCARE nurse.

Across NHs, the intervention elements’ perceived feasibility was high: 79% for STOP&WATCH, 85% for ISBAR, and 83% for the INTERCARE nurse. Again, NH 11 produced the lowest ratings for all intervention elements (STOP&WATCH: 52%; ISBAR: 63%; INTERCARE nurse: 64%). In contrast, NH2’s staff gave the highest respective ratings, at 100, 92 and 96%.

#### Uptake of intervention elements

Overall, the mean uptake levels were relatively high among NHs (STOP&WATCH [78%], ISBAR [77%], INTERCARE nurse [83%/ 77% nurse aids/RNs & LPNs]). Yet, there is a considerable inter-NH variability; for example, the uptake of STOP&WATCH varies from 64% (in NH6) to 100% (in NHs 2 and 4). Across NHs, variation regarding ISBAR and INTERCARE nurse uptake is also relatively high, ranging from 53 to 91% for ISBAR and 60–100% for INTERCARE nurse. Detailed results for all NHs regarding the acceptability, feasibility and uptake ratings for the three intervention elements are presented in Table 4.

**Table 4:** Results for nursing homes regarding the acceptability, feasibility and uptake ratings of the intervention elements

	STOP&WATCH						ISBAR						INTERCARE nurse							
	Acceptability^**a**^		Feasibility^**a**^		Uptake ^**c**^		Acceptability^**b**^		Feasibility^**b**^		Uptake ^**d**^		Acceptability^**a**^		Feasibility^**a**^		Uptake ^**e,f**^			
	RNs/LPNs, nurse aides		RNs/LPNs, nurse aides		Nurse aides		RN/LPNs		RN/LPNs		RN/LPNs		RN/LPNs		RN/LPNs		Nurse aides		RN/LPNs	
	% (n)		% (n)		% (n)		% (n)		% (n)		% (n)		% (n)		% (n)		% (n)		% (n)	
NH 1	70^7^	(26)	84^7^	(31)	−^7^	–	92	(33)	97	(35)	91	(32)	92	(34)	97	(36)	- ^7^	–	87	(28)
NH 2	86	(24)	100	(28)	100	(12)	**62**	(8)	92	(12)	90	(9)	89	(24)	96	(25)	80	(8)	90	(9)
NH 3	71	(55)	77	(57)	96	(24)	77	(36)	75	(35)	80	(40)	72	(54)	75	(55)	94	(17)	82	(32)
NH 4	100	(24)	100	(23)	100	(10)	94	(15)	94	(15)	**67**	(6)	100	(23)	100	(22)	100	(6)	75	(6)
NH 5	**64**	(35)	81	(43)	70	(16)	72	(18)	87	(21)	91	(23)	79	(41)	83	(43)	83	(15)	96	(24)
NH 6	**57**	(40)	76	(50)	**68**	(13)	**68**	(28)	87	(34)	86	(36)	**67**	(42)	70	(43)	87	(13)	74	(25)
NH 7	84	(38)	86	(38)	82	(14)	84	(21)	87	(21)	79	(29)	89	(39)	84	(36)	86	(12)	72	(21)
NH 8	74	(50)	85	(57)	**64**	(18)	76	(28)	89	(33)	73	(37)	74	(50)	79	(53)	81	(21)	84	(32)
NH 9	**54**	(26)	**62**	(30)	79	(11)	72	(23)	87	(28)	**53**	(19)	72	(33)	80	(36)	100	(13)	**62**	(24)
NH 10	**60**	(30)	70	(35)	75	(9)	74	(26)	88	(30)	**61**	(26)	72	(36)	84	(42)	73	(8)	**69**	(24)
NH 11	**26**	(14)	**52**	(27)	**68**	(13)	**44**	(14)	**63**	(19)	73	(30)	**62**	(33)	**64**	(34)	**61**	(11)	**60**	(18)
**Total**	**68**	(362)	79	(419)	78	(140)	74	(250)	85	(283)	77	(230)	79	(409)	83	(425)	83	(124)	77	(232)
***P*** value*	< .0001		< .0001		.043		.0021		0.0129		.0179		.0011		.0006		.636		.0359	

^a^STOP&WATCH and INTERCARE nurse acceptability and feasibility are calculated based on the full sample (i.e., registered nurses; licensed practical nurses, nurse aides)

^b^ISBAR acceptability and feasibility was calculated based on subset (i.e., registered nurses; licensed practical nurses)

^c^Uptake of STOP&WATCH was calculated for the sample of nurse aides based on one question;

^d^Uptake of ISBAR was calculated for the sample of RNs/LPNs based on one question;

^e^Adoption of INTERCARE nurse was calculated for the sample of nurse aides based on one question;

^f^Uptake of INTERCARE nurse was calculated for RNs/LPNs based on a scale with four questions;

^g^Nurse aides did not participate in the data collection due to language barriers; ^*^Chi-square; *P*-value: < 0.05; Values below 70% are bolded

*RN* Registered nurse, *LPN* Licensed practical nurse

### Qualitative data

#### Description of the implementation processes

The inter-NH implementation process was described using Mayring’s content analysis approach [27]. We compared one NH with a relatively high uptake, NH 2 (STOP&WATCH 100%, ISBAR 90%, INTERCARE nurse 80 and 90%), with NH 11 with a relatively lower uptake of the intervention elements, (STOP&WATCH 68%, ISBAR 73%, INTERCARE nurse 61 and 60%,), respectively. Overall, NH 2 is a stand-alone organization with 72 beds and one INTERCARE nurse. NH 11, on the other hand is part of a bigger group of multiple sites centrally managed, a total of 125 beds and two INTERCARE nurses working jointly. A detailed description of implementation processes in both NHs is displayed in Table 5.

**Table 5:** Description of Implementation Processes in Two Nursing Homes

	Example 1 (NH2)	Example 2 (NH11)
Internal project lead	INTERCARE nurse	The two INTERCARE nurses and the nursing director
Implementation plan	A written implementation plan and process description how to use STOP&WATCH, ISBAR & role description of INTERCARE nurse were developed	No implementation plan specific for this NH was developed
Involvement of care workers	Care workers were invited to provide feedback to adapt the processes to their needs	No involvement of care workers to adapt the processes
General introduction of project	A single information event for relatives, residents, physicians and the staff	A single information event for staff only
Information provision on individual units	The INTERCARE nurse was present at each unit’s team meeting to inform about the tools and own role using project educational materials	No information available
Supervision of implementation	Each unit appointed a champion that received a 2-day training on the instruments by an INTERCARE nurse	Regularly changing daily ward supervisors (often LPN) with no specific training
Tailoring implementation	INTERCARE nurse was fully supported by the nursing director having flexibility in implementing intervention elements and structuring clinical work	INTERCARE nurses were not supported by the nursing director and had no flexibility in implementing or structuring their clinical work
INTERCARE nurse start	Several months before the implementation	Concurrent with implementation
Role implementation	On units with high fluctuation, INTERCARE nurses spent additional time to maintain implementation	Due to high fluctuation on several units, INTERCARE nurses were delegated to work as registered nurses on the units, and they could not execute their roles as INTERCARE nurses
Evaluation and adaptation of processes	The NH upper management, unit managers, and INTERCARE nurse met regularly to discuss implementation. Use of the tools was documented and evaluated continuously. Champions elicited feedback from care workers` and discussed it with INTERCARE nurse in regular meetings to adapt processes	The nursing director and INTERCARE nurses met regularly with the management of the NH group to discuss implementation, yet no adaptations of the processes were made

*Note* Implementation processes described based on the analysis of structured discussion notes from in-person meetings with nursing home leadership and phone calls with INTERCARE nurses

#### Focus groups

Overall, 108 care workers (91% female; 50 nursing aides/ 34 RNs/ 24 LPNs) participated in 22 focus groups. Four distinct themes were developed based on the analysis: *being informed in due time*; *feeling supported in daily practice*; *fitting right in or requiring adaptation*; and *seeing the value or remaining skeptical.*

##### Being informed in due time

Care workers emphasized that receiving comprehensive information shortly before the implementation start reduced their uncertainty and stress while motivated them to tackle implementation challenges. The care workers who perceived that they were well-informed about and motivated regarding the intervention elements’ implementation reported to be informed in two ways. The first of these involved participation in a general information meeting before the intervention started. At this meeting, along with an overview of the intervention elements, participants learned what they could anticipate. Additionally, participants in some NHs received detailed information at the implementation start. During team meetings participants said they could ask questions relating to the intervention implementation and this helped them to clarify their concerns. Those who only attended the information meeting, which was held weeks before the implementation started, felt insufficiently informed. The care workers said it led to the circulation of contradictory information. For some, this led to false expectations that engendered confusion and frustration.

“Before the INTERCARE nurse came, I think we should all have been informed. Not everyone was informed correctly. […] when I came back, suddenly I had a nursing expert. I did not even know that she was coming. […] I have the feeling that the reason for that is that we did not get any information from the unit managers” (NH11, RN)*.*

##### Feeling supported in daily practice

During the implementation’s first 6 months, care workers appreciated the continuous support from peers or superiors and INTERCARE nurses in daily practice; they considered support an essential motivator to use the intervention elements. Many participants valued peer reminders to use the instruments and feedback immediately after using them.

"I think it is also important that we [care workers] always draw attention to the instruments for the co-workers, especially at the beginning, until we get used to them" (NH3, RN)*.*

A small number of participants also acknowledged that inter-unit peer exchange helped them learn from their experiences. Participants talked about receiving daily support directly on the units from their supervisors. In two NHs, participants mentioned additional specially-trained champions who provided support in using the intervention elements.

Some participants also voiced negative remarks about the frequently changing supervisors responsible for the instruments´ implementation. They noted that they had limited time and did not seem particularly interested either in reminding staff to use the instruments or in addressing their questions. Furthermore, LPNs appointed as charge nurses, who primarily oversee and coordinate day-to-day care, reported feeling overwhelmed by the additional functions, as they had only limited experience and expertise.

In contrast, participants with access to champions reported feeling continuously well-supported and being asked about their opinions. Similarly, several believed that their unit managers’ negative attitudes towards the intervention elements affected the implementation adversely.

“When we hear from unit managers comments like "yes, hey, what do we need this for" [the interventions] and so on, then, of course, this attitude trickles down to the staff” (NH9, RN)*.*

Whatever their reaction to the other intervention components, care workers appreciated the INTERCARE nurses’ support, noting that these nurses’ constant presence and frequent checks helped bolster their confidence. Additionally, care workers mentioned that when the charge nurse could not address their questions and uncertainties, INTERCARE nurses were helpful in filling that gap.

"If I was unsure about STOP&WATCH, I asked the INTERCARE nurse directly, and he always explained it to me" (NH1, nurse aide)*.*

However, some care workers felt that, over the first 6 months the INTERCARE nurses’ support levels decreased. This negatively influenced care workers’ motivation to use instruments. Several mentioned that, at some point, the INTERCARE nurses were no longer regularly present on their units to check and support their ISBAR and STOP&WATCH use. Participants noted that staff shortages, high turnover, and concurrent projects might have impacted available time resources, leading to decreased support. One nurse noted a strong contrast between her experience with her current employer and another she remembered:

Here in this nursing home, I have actually noticed how the nursing home leadership supports the project—simply that the INTERCARE nurse can really do her job. Because it is not the same everywhere, in the other nursing home, later on, they did not have time to check on us” (PH4, RN)*.*

##### Fitting right in or requiring adaptation

Perceived ease of both using interventions and of adapting them to individual NHs’ needs and resources considerably facilitates their adoption. For example, the ISBAR instrument’s target users—RNs and LPNs—found it readily understandable and appreciated that its addition required few changes to their existing routine. Noting that it was always visible on their units and included a clear summary of its steps, they agreed that it is straightforward to use both while evaluating patients and while communicating with physicians.

With ISBAR, it is easy, you use it when you contact a physician; everything is written down on the piece of paper, and each person has the pocket version. I see it as a template. It lays out all the steps and makes sure that nothing is forgotten. (NH5, RN)

In contrast, nurse aides found the STOP&WATCH instrument challenging to use, as they said it required changes to their team workflows—although these were for communication and collaboration. Some found it difficult to understand when—or even why—they should use it; some forgot to take it with them to the residents’ rooms to document their observations; and once they had completed the form, finding an RN or LPN to deal with the filled-in instrument was difficult.

The workflow needs re-thinking; the workflow is different and we need to do everything different than before. But it is not always clear when to fill it in [STOP & WATCH], I always wonder, is it really important now or not? And sometimes, you just do not know if it is already documented or not. […] Finally, when I filled it in, I did not get any feedback and could not say anything to the resident. (NH6, nurse aide)

Regarding both the ISBAR and the STOP&WATCH, daily practice was adapted over time to facilitate their use. Participants emphasized that, as their units varied in size, focus (e.g., dementia care vs. general long-term care) and skill-mix, they needed to work out how to best use them in each specific context. Therefore, they appreciated first being asked about their views, then seeing adaptations that responded to those views. This gave them the feeling that they were taken seriously.

The champions are interested, and we work well together. They know us and know our needs. When we tell them what is not good, they talk with the INTERCARE nurse, and they immediately tell us how we will do it from now on. (NH2, nurse aid)

##### Seeing the value or remaining skeptical

Most participating care workers were initially skeptical about the interventions’ relevance to practice and the additional workload it would entail. The characteristic that lessened their skepticism and facilitated utilization was discernable usefulness. This developed through successful clinical encounters (e.g., finding they could manage acute situations), self-reflection (nurse aides noticing that RNs took them seriously), exchanges with peers (e.g., sharing success stories), and direct feedback (e.g., physicians clearly appreciated RNs’ structured communication). Conversely, those who did not recognize these elements’ practical value did not want to use them.

For example, nurse aides’ initial resistance to STOP&WATCH changed as they became more aware of the changes they needed to look for, and as they saw that RNs and LPNs were taking their observations seriously and taking action. Several described this realization—that STOP&WATCH was a valuable communication tool - as a “eureka moment”. Regarding the ISBAR, RNs and LPNs described similar experiences. They described increasing confidence during conversations with physicians, leading to well-informed, timely decisions regarding residents’ care.

Regarding the INTERCARE nurse, most participants noted that they initially considered this role unnecessary. Over the first months of the intervention, though, they realized that the INTERCARE nurse’s kindness, support and coaching enabled them to respond more effectively to acute situations. This both reduced their decision-making burden concerning residents’ care and lightened their workloads. Such experiences motivated them to actively involve these nurses in daily situations. This sense of practical relevancy and influence increased the INTERCARE nurse’s utilization.

Before, I thought we would have extra work. Now I see we have instruments that are relevant for us and our work. And also, INTERCARE nurses are very helpful, and we can always call and ask them. (NH7, RN)

## Discussion

This study’s objective was to evaluate the implementation of three intervention elements to reduce hospitalizations in NH residents. Our mixed-methods study was embedded in a multicenter implementation study in eleven Swiss NHs [17]. Across NHs, our quantitative findings indicate considerable variations in the degree of acceptability, feasibility, and uptake of the intervention elements. The qualitative analyses highlight differences in the NHs’ internal implementation processes, along with a range of individual barriers and facilitators to implementation. In the following discussion, we clarify and discuss how the qualitative findings can explain differences in the quantitative results [20, 34].

### Complexity of interventions use

A consistent finding in our study were rather low levels of acceptability, feasibility and uptake regarding STOP&WATCH in comparison to the other two intervention elements (ISBAR, INTERCARE nurse). This issue in part can be explained by our qualitative findings. The theme “fitting right in or requiring adaptation” highlights the importance of an intervention’s perceived ease of use as a central precondition for its actual use. In case of STOP&WATCH, some nurse aides felt overwhelmed by the need to understanding instrument points, knowing and deciding when to fill it in and finally delivering it to an RN/LPN to evaluate the resident. This subjective complexity related to the number of individuals involved, the steps necessary to fulfil its purpose, and the lack of an immediate benefit for them often resulted in an initial reluctance towards using STOP&WATCH.

Previous studies exploring complexity-related challenges to the adoption of new processes could show almost linear relationships between increasing levels of complexity and overall implementation difficulty [22, 35, 36]. To facilitate implementation of particularly complex interventions, it has been recommended to examine perceived complexity early on to be able to adapt implementation strategies accordingly [35]. In this context the combination of implementation science methods with user-centered design approaches has been recommended in recent literature [37]. Potential strategies to address hidden complexity using user-centered design comprise conducting co-creation sessions or usability testing with future adopters, among others [38].

### Individual tailoring and adaptation processes

Based on our quantitative data we could identify several NHs that had overall rather low levels of acceptability and uptake of intervention elements as compared to others. When looking into the corresponding data on implementation processes it became apparent that several NHs did not individualize processes to the respective context of the units. Reflections of care workers during our focus groups support this missed opportunity for individualization. Some care workers felt ignored when proposing adaptations to the workflow. In contrast, care workers in other NHs were involved from the beginning in adapting processes related to the intervention elements to fit with their units’ workflow. It has been shown that early user involvement does not only result in higher acceptability and ultimately use of new interventions, but also increases commitment to the overall implementation [35]. For an intervention of this type, in addition to providing sufficient adaptation time, administrators need to consider inter-unit variation regarding organization (facility size, type of care provided), resources (e.g., workload, skill mix, turnover), and unique barriers to embedding proposed interventions within existing care processes [39].

### Leadership commitment to successful implementation

Our data on implementation processes clearly highlighted differences between the two NHs with the highest and lowest uptake. One central difference is the development of a written implementation plan and process description in collaboration with care workers in NH2, unlike the NH11. A second difference is the flexibility of INTERCARE nurses to divide their own time between implementation and clinical work. A further aspect that tells NH2 apart from NH11 and several other NHs is the appointment of champions to support the implementation directly on the units. These three aspects are indicative of a committed and transformational leadership at NH management level. Extensive research suggests that organizational leadership that creates a clear vision and fully commits to a project is an essential prerequisite for successful implementation [7, 15, 40, 41]. Furthermore, it has been shown that, anticipating potential difficulties when introducing new interventions and looking for feedback from staff to improve processes results in better staff buy-in and enhances implementation [15, 40, 41].

The disparity in the level of uptake between these exemplary NHs emphasizes the important role of champions in facilitating implementation. In the focus groups, care workers appreciated the comprehensive information and continuous daily support they received from the champions on the ward. Previous studies have established the value of continuous daily support by champions when implementing change [15, 42, 43]. Specifically, healthcare-related implementation studies have found significant associations between champions and implementation success [42, 44]. Furthermore, care workers in our focus groups emphasized the importance of committed and proactive unit managers to create a positive implementation climate. A considerable amount of literature has shown that middle managers’ willingness to act as role models, fill information gaps, solve problems on their units, and “sell” interventions, directly contributes to the intervention’s uptake [45, 46]. Hence, implementation projects in organizations like nursing homes, should strive for active involvement of middle managers, i.e. unit managers, to fuel implementation efforts.

### Strengths and limitations

Given the complexity of implementing interventions in the real world, this study highlights the value of using both qualitative and quantitative methods to assess implementation processes [12, 34]. The triangulation of three validated data sources including 22 focus group interviews, a survey of 573 care workers and implementation notes, followed by rigorous data analysis allowed us both to develop a rich understanding of the findings and to generate reliable recommendations for uptake into broader clinical practice.

Due to time and financial limitations, we were not able to transcribe all interviews. However, by listening repeatedly to all of the un-transcribed audio files, the first author was able to develop summary maps, which the entire research team then discussed. One further limitation is that, as we did not have a comparison group, we were unable to examine contrasts between care workers’ perspectives and acceptability, feasibility, and uptake of intervention elements in a control grouHowever, regarding our three original aims—we focused on understanding how to implement the three studied intervention elements, with a particular focus on implementation barriers and facilitators.

## Conclusions

Our analysis uncovered potentially modifiable barriers and facilitators. These findings can inform future implementation strategies, for example, “nominate and prepare champions”,” conduct educational meetings”, or “capture and share local knowledge”, to help enhance interventions’ routine uptake [14].

Within intervention teams, unit-level champions proved invaluable in closing information gaps, facilitating buy-in, and providing support. We also found that enlisting intervention users for implementation processes co-design boosted their motivation and engagement in the intervention. Other findings reinforce the importance of pre-emptively addressing use barriers such as intervention complexity by noting their influence on early practice patterns. The most successful strategy involved presenting the necessary elements in a simple, easily accessible format, while easing uptake via continuous support. These results are essential for implementation efforts and may be applied to other interventions and other NH contexts.

## Supplementary Information

**Additional file 1.** Supplementary material.

## Data Availability

Quantitative data supporting the findings of this study with a code book are available from the principal investigator (MS) upon request. The qualitative data analyzed during the current study are not shared publicly due to concerns that cannot be addressed through standard data depositing practices such as anonymizing data and the risk of re-identifying participants. However, the data is available upon reasonable request.

## Abbreviations

AACTT: Action, Actor, Context, Target, Time
AIM: Acceptability of Intervention Measure
CFIR: Consolidated Framework for Implementation Research
ERIC: Expert Recommendations for Implementing Change
FIM: Feasibility of Intervention Measure
INTERCARE: Nurse-led model of care in Swiss nursing homes: improving interprofessional care for better resident outcomes
ISBAR: Introduction, Situation, Background, assessment, Recommendation
LPN: Licensed practical nurse
NH: Nursing home
RN: Registered nurse

## References

[1] Graverholt B, Forsetlund L, Jamtvedt G (2014). Reducing hospital admissions from nursing homes: A systematic review. BMC Health Serv Res.

[2] Fogg C (2018). Hospital outcomes of older people with cognitive impairment: An integrative review. Int J Geriatr Psychiatry.

[3] James BD (2019). Cognitive decline after elective and nonelective hospitalizations in older adults. Neurology.

[4] Santosaputri E, Laver K, To T (2019). Efficacy of interventions led by staff with geriatrics expertise in reducing hospitalisation in nursing home residents: A systematic review. Australas J Ageing.

[5] Hutchinson AF (2015). A longitudinal cohort study evaluating the impact of a geriatrician-led residential care outreach service on acute healthcare utilisation. Age Ageing.

[6] Schippinger W (2012). mobile geriatric consultant services for rest homes. Study of the effects of consultations by internal medicine specialists in the medical care of rest home residents. Z Gerontol Geriatr.

[7] Ouslander JG (2014). The interact quality improvement program: An overview for medical directors and primary care clinicians in long-term care. J Am Med Dir Assoc.

[8] Rantz MJ (2017). Successfully reducing hospitalizations of nursing home residents: Results of the missouri quality initiative. J Am Med Dir Assoc.

[9] Blackburn J (2020). Reducing the risk of hospitalization for nursing home residents: Effects and facility variation from optimistic. J Am Med Dir Assoc.

[10] Ingber MJ (2017). Initiative to reduce avoidable hospitalizations among nursing facility residents shows promising results. Health Aff (Millwood).

[11] Peters DH, et al. Implementation research, how to do it. Sports Med. 2013:347:f6753. 10.1136/bmj.f6753. 10.1136/bmj.f6753 24259324

[12] Proctor E (2010). Outcomes for implementation research: Conceptual distinctions, measurement challenges, and research agenda. Adm Policy Mental Health Mental Health Serv Res.

[13] Pulst A, Fassmer AM, Schmiemann G (2021). Unplanned hospital transfers from nursing homes: Who is involved in the transfer decision? Results from the homern study. Aging Clin Exp Res.

[14] Powell BJ (2015). A refined compilation of implementation strategies: Results from the expert recommendations for implementing change (eric) project. Implement Sci.

[15] Li SA (2018). Organizational contextual features that influence the implementation of evidence-based practices across healthcare settings: A systematic integrative review. Syst Rev.

[16] Curran GM (2012). Effectiveness-implementation hybrid designs: Combining elements of clinical effectiveness and implementation research to enhance public health impact. Med Care.

[17] Zúñiga F (2019). Strengthening geriatric expertise in swiss nursing homes: Intercare implementation study protocol. J Am Geriatr Soc.

[18] Basinska K, et al. Registered nurses in expanded roles improve care in nursing homes: Swiss perspective based on the modified delphi method. J Adv Nurs. 2020;77(2) 10.1111/jan.14644. 10.1111/jan.14644 PMC7894469 33222269

[19] Zúñiga F, Guerbaai RA, De Geest S, Popejoy LL, Barkova J, Denhaerynck K, et al. Positive effect of the intercare nurse-led model on reducing nursing home transfers: A non-randomized stepped-wedge design. J Am Geriatr Soc. 2022; 10.1111/jgs.17677. 10.1111/jgs.17677 PMC9305956 35122238

[20] Creswell JW (2011). Best practices for mixed methods research in the health sciences.

[21] Presseau J (2019). Action, actor, context, target, time (aactt): A framework for specifying behaviour. Implement Sci.

[22] Damschroder LJ (2009). Fostering implementation of health services research findings into practice: A consolidated framework for advancing implementation science. Implement Sci.

[23] Weiner BJ (2017). Psychometric assessment of three newly developed implementation outcome measures. Implement Sci.

[24] Nuzzo RL (2019). Making continuous measurements into dichotomous variables. PM&R.

[25] Courtier N (2018). Active - a randomised feasibility trial study protocol of a behavioural intervention to reduce fatigue in women undergoing radiotherapy for early breast cancer: Study protocol. Pilot Feasib Stud.

[26] R Development Core Team, R (2018). A language and environment for statistical computing (version 3.5.1).

[27] Mayring P, Mey G, Mruck K (2010). Qualitative inhaltsanalyse in Handbuch qualitative forschung in der psychologie.

[28] Baxter R (2016). What methods are used to apply positive deviance within healthcare organisations? A systematic review. BMJ Qual Safety.

[29] Braun V, Clarke V (2006). Using thematic analysis in psychology. Qual Res Psychol.

[30] Braun V, Clarke V (2019). Reflecting on reflexive thematic analysis. Qual Res Sport Exerc Health.

[31] Lincoln YS, Guba EG, Denzin NK (1994). Competing paradigms in qualitative research, in Handbook of qualitative research. Competing paradigms in qualitative research, L.Y.

[32] Tessier S (2012). From field notes, to transcripts, to tape recordings: Evolution or combination?. Int J Qual Methods.

[33] VERBI Software (2018). Maxqda 2018.

[34] Palinkas LA (2011). Mixed method designs in implementation research. Adm Policy Ment Health.

[35] Grol R, Wensing M, Wensing M, Grol R, Grimshaw J (2020). Characteristics of successful innovations. Improving patient care: The implementation of change in health care.

[36] Rogers EM (1995). Diffusion of innovations.

[37] Dopp AR (2020). Aligning implementation and user-centered design strategies to enhance the impact of health services: Results from a concept mapping study. Implement Sci Commun.

[38] Dopp AR (2019). A glossary of user-centered design strategies for implementation experts. Transl Behav Med.

[39] Baker R (2010). Tailored interventions to overcome identified barriers to change: Effects on professional practice and health care outcomes. Cochrane Database Syst Rev.

[40] Sarkies M (2020). Avoiding unnecessary hospitalisation for patients with chronic conditions: A systematic review of implementation determinants for hospital avoidance programmes. Implement Sci.

[41] Vogelsmeier A (2021). Results of the missouri quality initiative in sustaining changes in nursing home care: Six-year trends of reducing hospitalizations of nursing home residents. J Nutr Health Aging.

[42] Miech EJ (2018). Inside help: An integrative review of champions in healthcare-related implementation. SAGE Open Med.

[43] Flodgren G (2019). Local opinion leaders: Effects on professional practice and healthcare outcomes. Cochrane Database Syst Rev.

[44] Birken SA, Currie G (2021). Using organization theory to position middle-level managers as agents of evidence-based practice implementation. Implement Sci.

[45] Birken S (2018). Middle managers' role in implementing evidence-based practices in healthcare: A systematic review. Implement Sci.

[46] Aarons GA, Ehrhart MG, Farahnak LR (2014). The implementation leadership scale (ils): Development of a brief measure of unit level implementation leadership. Implement Sci.

[47] A., World Medical (2013). World medical association declaration of helsinki: Ethical principles for medical research involving human subjects. JAMA.

