# Supplementary material for: Implementation of a complex intervention to reduce hospitalizations from nursing homes: a mixed-method evaluation of implementation processes and outcomes
Source: BMC Geriatr. 2022 Mar 12;22:196. doi: 10.1186/s12877-022-02878-y (PMC8918313; doi:10.1186/s12877-022-02878-y)

**Supplementary material**

**Supplementary file 1.** *Questions Asked to Evaluate The Uptake Of Three Intervention Elements For The Professional Group*

**Supplementary file 2.** *Six Steps of Reflexive Thematic And The Authors Role*

**Supplementary file 3.** *STOP&WATCH instrument*

**Supplementary file 4.** *ISBAR Instrument*

**Supplementary file 1.**

*Questions Asked to Evaluate the Uptake of Three Intervention Elements for the Professional Group*

| **Nurse aides** | **Registered nurses / licensed practical nurses** |
| --- | --- |
| **STOP&WATCH** | **ISBAR** |
| I use STOP&WATCH when I notice a difference in a resident's condition | I use ISBAR when I contact a physician |
| **INTERCARE nurse** | **INTERCARE nurse** |
| When required I receive coaching and support for the resident's care | When required I receive coaching and support for the resident's care |
|  | When required I receive coaching and support in conversation with residents/relatives |
|  | When required I receive support in the decision-making process about residents’ care |
|  | When required I receive coaching and support in preparation for physician visits/communication |

**Supplementary file 2.**

*Six Steps of Reflexive Thematic and The Authors Role [25, 26]*

| **Steps** | **Description** |
| --- | --- |
| 1. Familiarizing with the data | For data familiarization, KB read through eight transcripts and listened to the remaining 14 audio files. While reading the transcript, the KB took notes in chunks and for the audio files, KB took notes in a qualitative diary about interesting parts of data. |
| 1. Generating initial codes | After that, KB started inductive coding of eight transcripts one by one. After rounds of coding, the codes were reviewed and compared with the coded text by KB and discussed with TB. In the next steps, codes were applied to the audio files, and meaningful text passages were transcribed. New codes were added for meaningful data when needed. Additionally, for all audio files, summaries of transcripts as thematic maps were created. |
| 1. Generating themes | Based on the codes from transcripts and audio files, KB developed the first summary of themes and discussed them with TB and FZ. |
| 1. Reviewing themes | KB revived the themes during an iterative back-and-forth process and compared them against raw data (transcripts, audio files when necessary) and summaries of audio files. After each round, a thematic map was adopted by KB and discussed with TB and FZ until they collectively agreed on meaningful patterns of theme. After each discussion round, KB went back to the transcripts and audio files to revise themes. As typical for thematic analysis, we adapted reviewed themes several times before deciding upon a clear thematic structure. |
| 1. Defining and naming themes | Generated themes, including illustrative quotes, were presented and discussed during a meeting with all authors, presented to the intervention nursing homes. No adaptations were needed. |
| 1. Producing the report | A written description of themes with carefully selected quotes was written by KB and reviewed by all co-authors (FZ, MS, SDG, DN, RAG, NIHW, TB) |

**Supplementary file 3.**

*STOP&WATCH instrument*


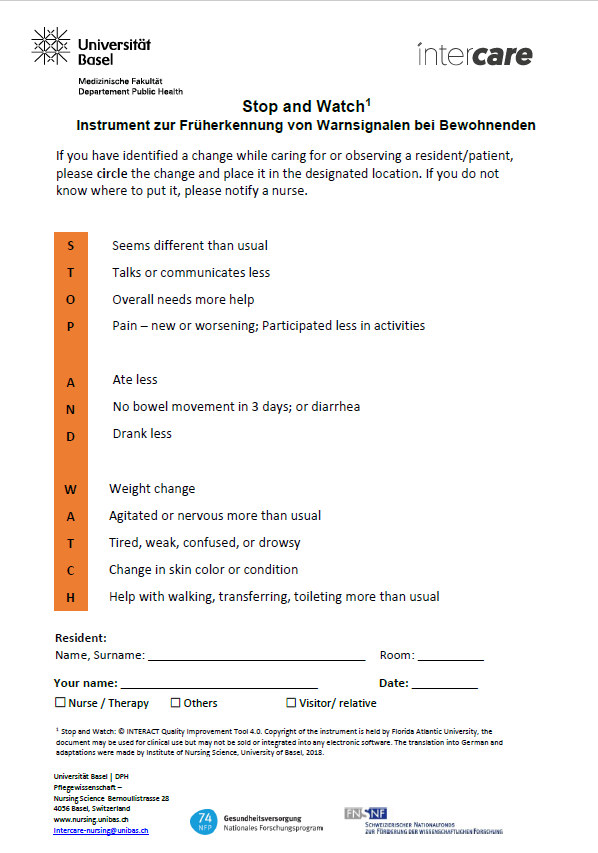


**For early recognition of changes in residents condition**

**Supplementary file 4.**

*ISBAR Instrument*


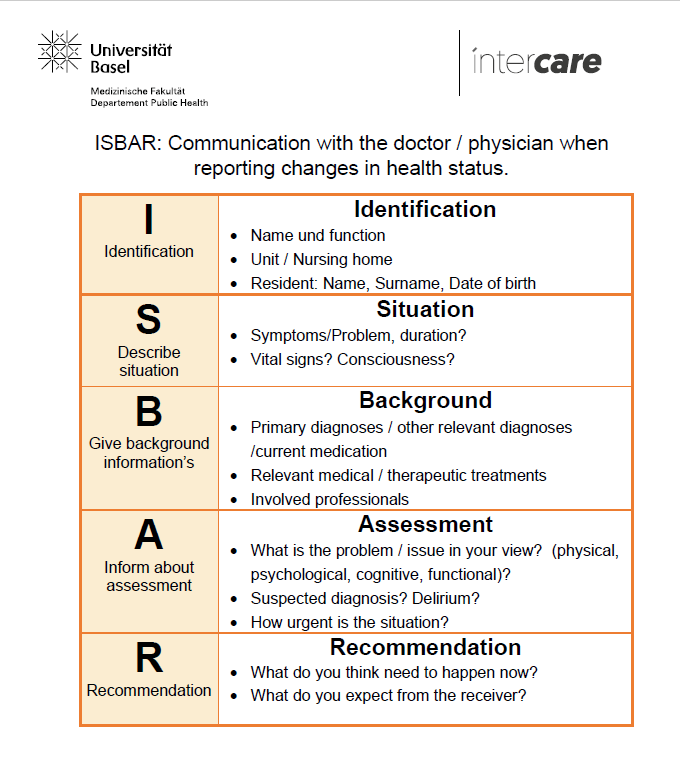

Supplement: Supplementary file 1 — Additional file 1. Supplementary material. [file 12877_2022_2878_MOESM1_ESM.docx]
